# Notes on the Nodes Found in the Lungs Caused by Actinomyces Bovis, Micrococcus Botryogenus, Strongylus, Echinococci, and Aspergillus

**Published:** 1896-06

**Authors:** J. T. Glennon

**Affiliations:** Student in New York College of Veterinary Surgeons


### NOTES ON THE NODES FOUND IN THE LUNGS, CAUSED BY ACTINOMYCES BOVIS, MICROCOCCUS BOTRYOGENUS, STRONGYLUS, ECHINOCOCCI, AND ASPERGILLUS.

BY J. T. GLENNON,
STUDENT IN NEW YORK COLLEGE OF VETERINARY SURGEONS.

*Actinomycosis.* The germs causing this disease are termed actinomyces bovis; they penetrate the organism in various ways, but usually through the digestive tract. Infection of the lungs is generally secondary to a lesion of the jaw. In the lungs the lesions may resemble miliary tubercles. Oftenest, however, the lesions are soft, have a sarcomatous aspect, and a reddish-yellow color. At times they have the density of a fibroma, and are of a whitish-gray color, and again they are of a spongy consistency and are formed of connective-tissue stroma, in which are enclosed numerous nodules of various dimensions, from the size of a millet-seed to a pea. Nodules are generally encapsulated, and on section the masses of germs appear as sulphur-yellow spots. Several tumors may become confluent and form small tuberculiform masses the size of an egg or larger. The actinomycoma may also undergo purulent destruction and later form a cavity.

*Botryomycosis.* The parasites producing this disease were discovered by Prof. Bollinger, in 1869, in the lungs of a horse; in the lungs the infection is usually secondary. Grayish-white nodules are formed, from the size of a millet-seed up to that of a walnut; they are of a fibrous nature, hard and sharply circumscribed. On section the centres of these nodes are always found broken down. The capsule is much thicker and the contents more fibrous than is the case with the nodules caused by actinomyces bovis. In botryomycosis the nodes uniting with one another may form very large abscesses containing a slimy substance mixed with pus, and on evacuation of this material cavities remain.

*Pulmonary strongylosis.* This disease in a nodular form is due to ova deposited in pulmonary alveoli. There are small, rounded centres about the size of miliary tubercles or even as large as a small pea, yellowish-white or sometimes dark red, and placed for the most part at the periphery of the lungs close under the pleura. They may all undergo cheesy or calcareous degeneration, but are not easily enucleated.

*Echinococci.* Echinococci in the lungs of cattle may undergo cheesy degeneration, and then resemble tubercles closely. They appear as areas of thick, mortarlike, yellow, cheesy matter, are contained in white capsules, and are therefore easily enucleated. The size is variable, from that of a small seed upward.

*Aspergillus.* Pulmonary aspergillus assumes the character of purulent follicular pneumonia. Follicles vary in size from a hempseed to a pea. Ordinarily they exist in large numbers and are disseminated throughout the entire lung and pleura. They may also become confluent and consist of a connective-tissue capsule, which is filled with pus containing fungi and separated from healthy tissue by a hepatized zone. Rarely there may be present a well-marked, diffuse pneumonia, characterized by hepatization and inflammatory infiltration of the interlobular connective-tissue (Röckel). This condition bears a striking anatomical similarity to contagious pleuro-pneumonia. Microscopic examination is necessary to insure correct diagnosis.

